# Melatonin Inhibits Embryonic Salivary Gland Branching Morphogenesis by Regulating Both Epithelial Cell Adhesion and Morphology

**DOI:** 10.1371/journal.pone.0119960

**Published:** 2015-04-15

**Authors:** Aya Obana-Koshino, Hitomi Ono, Jiro Miura, Manabu Sakai, Hitoshi Uchida, Wataru Nakamura, Kanji Nohara, Yusuke Maruyama, Atsuhiko Hattori, Takayoshi Sakai

**Affiliations:** 1 Department of Oral-facial Disorders, Osaka University Graduate School of Dentistry, Suita, Osaka, Japan; 2 Division for Interdisciplinary Dentistry, Osaka University Graduate School of Dentistry, Suita, Osaka, Japan; 3 Clinical Laboratory, Osaka University Dental Hospital, Suita, Osaka, Japan; 4 Laboratory of Oral Chronobiology, Osaka University Graduate School of Dentistry, Suita, Osaka, Japan; 5 Department of Biology, Tokyo Medical and Dental University, Ichikawa, Chiba, Japan; NCMLS, Radboud University Nijmegen Medical Center, NETHERLANDS

## Abstract

Many organs, including salivary glands, lung, and kidney, are formed by epithelial branching during embryonic development. Branching morphogenesis occurs via either local outgrowths or the formation of clefts that subdivide epithelia into buds. This process is promoted by various factors, but the mechanism of branching morphogenesis is not fully understood. Here we have defined melatonin as a potential negative regulator or “brake” of branching morphogenesis, shown that the levels of it and its receptors decline when branching morphogenesis begins, and identified the process that it regulates. Melatonin has various physiological functions, including circadian rhythm regulation, free-radical scavenging, and gonadal development. Furthermore, melatonin is present in saliva and may have an important physiological role in the oral cavity. In this study, we found that the melatonin receptor is highly expressed on the acinar epithelium of the embryonic submandibular gland. We also found that exogenous melatonin reduces salivary gland size and inhibits branching morphogenesis. We suggest that this inhibition does not depend on changes in either proliferation or apoptosis, but rather relates to changes in epithelial cell adhesion and morphology. In summary, we have demonstrated a novel function of melatonin in organ formation during embryonic development.

## Introduction

Melatonin (N-acetyl-5-methoxytryptamine) is a hormone secreted by the pineal gland. It is synthesized from L-tryptophan by the sequential actions of four enzymes: tryptophan hydroxylase, 5-hydroxytryptophan amino acid decarboxylase, arylalkylamine N-acetyltransferase (AANAT), and hydroxyindole-O-methyltransferase (HIOMT) [1]. Its secretion into the blood is controlled by the endogenous circadian clock, the suprachiasmatic nucleus (SCN), and is regulated by environmental light. Termed the “hormone of darkness,” it is secreted in darkness in both day-active (diurnal) and night-active (nocturnal) animals [2]. Previous research has shown that the membrane-bound melatonin receptors, MT1 and MT2 [3,4], are expressed in the central nervous system [5] and control both circadian rhythms and sleep. Both MT1 and MT2 receptors are widely expressed in many peripheral organs as well as in the central nervous system. *MT1* mRNA was identified in the heart, kidney, liver, and lung, and *MT2* mRNA was found in both the lung and thymus in mice [6,7]. Thus, MT1 and MT2 receptors are discretely distributed in areas of peripheral target organs.

Melatonin is also produced by various peripheral organs, tissues, and cells, including the ovary, gastrointestinal tract, bone marrow, and lymphocytes [7,8]. Usually, the melatonin concentration in these organs/cells is much higher than that in blood. Previous studies have shown that melatonin is present in saliva as well as in serum [9,10]. It was thought that melatonin entered the saliva through passive diffusion from the blood [11,12]. However, a recent study demonstrated the expression and localization of AANAT in the ductal cells of both adult rat and human salivary glands. And the potent melatonin receptor, MT1 expressed in rat buccal mucosa [8]. These findings suggest that melatonin is produced and secreted by adult salivary glands and has some function in the oral cavity via MT1. Melatonin has various physiological functions [13], including regulating the circadian rhythm [14], free-radical scavenging [15], antioxidation, the immune system [16], antitumor activity [17], body temperature [18], hormone secretion influencing sexual development [19], bone formation [20], and tooth development [21]. Its many biological functions and wide distribution imply that melatonin has a physiological function in the salivary gland as well as in other tissues and organs. Recent research showed melatonin affects mouse gonadal development [1]. However, there is no information on its role in the embryo. The function of melatonin in embryonic organs such as the salivary gland during embryonic development also has not been investigated.

The mouse salivary gland starts as a single epithelial bud surrounded by mesenchyme at embryonic day 12 (E12). The branching process is initiated at E12.5 by the formation of shallow clefts that deepen and subdivide the epithelium into multiple buds [22]. Early branching is not dependent on cell proliferation but is dependent on multiple process, such as cell-cell interaction, cell-matrix interaction, and cell-shape changes [23]. The extracellular matrix (ECM) protein fibronectin has been identified as an early cleft initiator acting either directly or indirectly by modulating cell-cell adhesive interactions via E-cadherin localization [24]. Subsequent repetitive cycles and bud/duct elongation allow the developing glands to form highly branched structures by E14 to E15. The branching continues over the subsequent days of embryonic development. Branching morphogenesis also occurs in the lung, kidney, mammary, and lacrimal glands [25]. This process involves coordinated cell growth, proliferation, migration, apoptosis, and epithelial-mesenchymal cell interactions. However, the mechanism of branching morphogenesis is poorly understood in these organs as well.

Previous studies have shown that ECM molecules [24] and growth factors [26] play an important role in gland development. In recent studies, growing evidence suggests that the physical microenvironment and growth factors direct cell fate in developing tissues [27,28]. Therefore, we hypothesized that not only growth factors but also local hormones, such as melatonin, may be involved in organ formation in the microenvironment. This study is the first report to show that melatonin affects the morphogenesis of an embryonic organ.

## Materials and Methods

### Submandibular gland culture

All animal experiments were carried out in strict accordance with the recommendations in the Guide for the Care and Use of Laboratory Animals of the National Institutes of Health. The protocol was approved by the Committee on the Ethics of Animal Experiments of Osaka University Graduate School of Dentistry, Japan (Permit Number: 25-004-0). All surgeries were performed under sodium pentobarbital anesthesia, and all efforts were made to minimize suffering. Submandibular glands (SMGs) dissected from both embryonic and postnatal ICR mice (Japan SLC, Inc., Hamamatsu, Japan) were cultured on Nucleopore membranes (1 μm pore size, GE Healthcare UK Ltd., Buckinghamshire, England) in serum-free Dulbecco’s modified Eagle’s medium/F12 medium, as previously described [26]. E12.5 SMGs were cultured with either 1 μM or 100 μM melatonin, 10 μM luzindole (LZ), and 100 μM 6-hydroxymelatonin (6-OHMel) (Sigma-Aldrich Corporation, St. Louis, MO, USA). Melatonin, LZ, and 6-OHMel was dissolved in ethanol (final ethanol concentration of <1%) and diluted with phosphate-buffered saline (PBS) to the desired melatonin concentration. Control glands were cultured with an equal volume of the vehicle ethanol.

### Quantitative real-time reverse transcription-polymerase chain reaction

SMGs were dissected from E12.5, 14, 15, 16, postnatal day 1 (P1) and 12 ICR mice. Total RNA was isolated from both embryonic and postnatal tissues using TRIzol Reagent (Life Technologies, Carlsbad, CA, USA) according to the manufacturer’s instructions, and was treated with DNase I (Roche Applied Science, Penzberg, Upper Bavaria, Germany) to avoid genomic DNA contamination. For cDNA synthesis, reverse transcription was performed using the PrimeScript RT Reagent Kit (Takara Bio Inc., Otsu, Shiga, Japan). Quantification of PCR products was performed using the MyiQ Single-Color Real-Time PCR Detection System (Bio-Rad Laboratories, Inc., Hercules, CA, USA) with iQ SYBR Green Supermix (Bio-Rad Laboratories, Inc., Hercules, CA, USA). The amplification program comprised 40 cycles of denaturation at 95°C for 5 s, annealing at 55°C for 20 s, and extension at 72°C for 20 s. The qPCR results for each sample were normalized by *glyceraldehyde-3-phosphate dehydrogenase* (*Gapdh*). The results were expressed as normalized ratios, and experiments were repeated three times. The primer sequences used were as follows:

*MT1*: 5′-CTACGTGTTCCTGATATGGATGCT-3′ and 5′-ACTGGAGTGTTCCGGTTTGC-3′; *Gapdh*: 5′-CCATCACCATCTTCCAGGAG-3′ and 5′-GCATGGACTGTGGTCATGAG-3′; *E-cadherin*: 5′-ACGTATCAGGGTCAAGTGCC-3′ and 5′-CCTGACCCACACCAAAGTCT-3′.

### Immunofluorescence

Paraffin-embedded E12.5 SMGs were evaluated. Tissue sections were deparaffinized and antigen retrieval was performed by autoclave heating (instant antigen retrieval H buffer, 121°C for 5 min). The slides were washed in PBS. The samples were first incubated with M.O.M. Mouse Ig Blocking Reagent (Vector Laboratories, Inc., Burlingame, CA, USA) and then with primary antibodies in diluent (1× PBS, containing 8% protein concentrate; M.O.M. Kit; Vector Laboratories, Inc.) for overnight at room temperature. Specific antibodies were used against E-cadherin (dilution 1:100; BD Biosciences, Franklin Lakes, NJ, USA) and against the melatonin receptor type 1A (dilution 1:100; Assay Biotechnology Co., Inc., Sunnyvale, CA, USA). After washing with PBS containing 0.5% Tween 20 (PBST), the tissues were incubated with either Cy3-labelled donkey anti-mouse or Cy5-labelled donkey anti-rabbit IgG for 2 h at room temperature (dilution 1:100; Life Technologies, Carlsbad, CA, USA) in diluent (5% donkey serum, containing 8% protein concentrate). The tissues were then washed in PBS and incubated with Dapi (dilution 1:500; Thermo Scientific, MA, USA).The coverslips were mounted on glass slides by inverting them into mounting solution, ProLong Gold antifade reagent (Life Technologies, Carlsbad, CA, USA) and examined by confocal microscopy (Laser Scanning Microscope SP8; Leica microsystems, Wetzlar, Germany). Immunostaining was repeated at least three times.

### Detection of cell proliferation and apoptosis

E12.5 SMGs were cultured in medium either with or without melatonin for 48 h. Cell proliferation was measured using a Click-iT EdU Alexa Fluor Imaging Kit (Life Technologies, Carlsbad, CA, USA) following the commercial protocol. SMGs were incubated with 10 μM EdU at 37°C for 3 h. They were fixed in 3.7% formaldehyde for 15 min, washed twice with 3% bovine serum albumin in PBS, incubated with 0.5% Triton X-100 in PBS for 20 min, and then the Click-iT (Life Technologies, Carlsbad, CA, USA) reaction cocktail was added at 37°C for 30 min. Apoptosis was detected by TUNEL using an In Situ Cell Death Detection Kit (TMR-red; Roche Applied Science, Penzberg, Upper Bavaria, Germany) following the commercial protocol. SMGs were fixed with 2% paraformaldehyde for 60 min, permeabilized with 0.1% Triton X-100 in 0.1% sodium citrate PBS on ice for 2 min, and incubated with TUNEL reaction mixture at 37°C for 1 h. The SMGs were also stained with peanut lectin conjugated to Alexa Fluor 647 (Vector Laboratories, Inc.) to stain the epithelial cells. Immunofluorescence was examined using confocal microscopy (LSM 510; Carl Zeiss AG). The fluorescent pixels from all optical sections of each SMG were measured, and the data were expressed as a ratio of the total pixel area of the gland using the ImageJ software (National Institutes of Health, Bethesda, MA, USA). At least five SMGs per condition were used for quantification, and the experiments were repeated three times.

### Western blot analysis

E12.5 SMGs were cultured in medium either with or without melatonin for 60 h. SMGs were lysed with RIPA buffer (Nacalai Tesque, Kyoto, Japan) supplemented with protease and phosphatase inhibitors (Nacalai Tesque, Kyoto, Japan). Cell lysates were subjected to centrifugation (15.000× g) for 10 min, and the supernates were heated at 95°C for 5 min in denaturing Laemmli buffer (Bio-Rad Laboratories, Inc., Hercules, CA, USA). Proteins were separated by SDS-PAGE and transferred to Polyvinylidene difluoride (PVDF) membranes (Bio-Rad Laboratories, Inc., Hercules, CA, USA). The membranes were blocked with 3% low fat milk in Tris-buffered saline with Tween 20 and then incubated with anti-alpha tubulin (1:2000; Abcam. Cambridge, UK) and anti-E-cadherin (dilution 1:1000; Cell Signaling Technology, Boston, USA). The bound antibodies were detected with anti-rabbit IgG, HRP-linked antibody (dilution 1:1000; Cell Signaling Technology, Boston, USA) and the ECL detection reagent (Bio-Rad Laboratories, Inc., Hercules, CA, USA). The band intensity was quantified using ImageJ software.

### Transmission electron microscopy

E12.5 SMGs were cultured in medium either with or without melatonin for 48 h. SMGs were fixed with modified Karnovsky’s fixative containing 2.5% glutaraldehyde and 2% paraformaldehyde in 0.1 M cacodylate buffer at pH 7.4 for 2 h, as previously described [29]. Then, the tissues were rinsed in cacodylate buffer at 4°C for 15 min. Postfixation was performed in 2% osmium tetroxide in 0.1 M cacodylate buffer for 2 h. The tissues were dehydrated with a series of increasing ethanol concentrations and embedded in an epoxy resin mixture (Quetol812, Nissin EM, Tokyo, Japan). Trial sections (2 μm thick) were cut and counterstained with toluidine blue to observe the morphology of the SMGs using an optical microscope. Ultrathin sections were cut using diamond knives, and the fine sections (70 nm thick) were mounted on copper 100 mesh grids. The grids were stained with both 24% uranyl acetate and 0.4% lead citrate and examined using an H-800 transmission electron microscope (Hitachi Ltd., Chiyoda, Tokyo, Japan) adjusted to 200 kV.

Immuno-electron microscopy was performed according to the following protocol. SMGs were fixed with 2% PFA and 0.3% glutaraldehyde, and embedded in LR Gold Resin (Electron Microscopy Sciences, PA, USA) at -20°C with an ultraviolet polymerizer system (DOSAKA EM CO. LTD., Kyoto Japan) for 24 h. Then, the samples were sectioned to 90 nm in thickness and mounted on nickel grids (Nisshin EM, Tokyo, Japan) in an ultramicrotome (Ultrome V, LKB, Sweden). The sections were immersed in blocking solution (4% BSA) and incubated 3 h at room temperature with rabbit monoclonal E-cadherin (dilution 1:30, Cell Signaling Technology, Boston, USA). Secondary antibody, donkey anti-rabbit IgG (18 nm colloidal gold; Jackson, PA, USA), was diluted (1:20) containing 0.2% tween 20 and 5% albumin in tris-buffered saline. Specimens were fixed with 2% glutaraldehyde and then stained with 2% uranylacetate. A transmission electron microscope at 200 keV was employed for detection of E-cadherin-bound colloidal golds.

### Expression of melatonin in submandibular glands and culture medium

In this study, C3H/HeN mice (Japan SLC, Inc., Hamamatsu, Japan), in which the presence of melatonin has been reported [30], were used to determine whether embryonic SMGs produce melatonin. The amount of melatonin in eight E12.5 SMGs and in one adult SMG was measured at least 5 times. Eight E12.5 SMGs were grown for 12 or 24 h, and the amount of melatonin in the culture medium and in the SMGs was measured at least five times. The amount of melatonin in these samples was determined using methods described previously [31]. Pooled SMGs in 1 ml of distilled water with 50 μl of 1.4 mM butylated hydroxytoluene were extracted with 10 ml of chloroform for 24 h at 10°C, and the chloroform phase was evaporated to extract the melatonin. For isolation of melatonin from the culture medium, 0.15 ml of culture medium was added to 0.85 ml distilled water, and then 4 ml of chloroform was added, and the mixture was stirred. The chloroform phase was evaporated. The extracts were redissolved in 300 μl of HPLC mobile phase solution, comprising 50 mM ammonium acetate and 30% methanol (vol/vol), and adjusted to pH 4.8 with acetic acid. After centrifugation at 500 × *g* for 1 min at room temperature, the supernate was filtered through a Millex-LH Filter Unit 0.45 μm (Millipore Corporation, Bedford, MA, USA) and subjected to chromatography using a Capcell Pak C18 MG-II 5 μm column (4.6 × 250 mm; Shiseido Co., Ltd., Tokyo, Japan) and an RF-10AXL Spectrofluorometric Detector (Shimadzu Corporation, Kyoto, Japan). The detector was operated at an excitation wavelength of 280 nm and an emission wavelength of 340 nm. All separations were performed isocratically at a mobile phase flow rate of 0.8 ml/min and at 40°C. The peaks in the chromatograms were identified by comparing the retention time with that of both standard melatonin and structurally-related indole compounds. Melatonin levels were determined by the measurement of each peak area. The limit of sensitivity of the assay was as low as 1 pg for a 2:1 signal-to-noise ratio. The intra- and inter-assay variation coefficients were 0.36% (*n* = 5) and 0.8% (*n* = 7), respectively. Melatonin and structurally-related indoles were obtained from Sigma-Aldrich Corporation (St. Louis, MO, USA).

### Statistical analysis

Statistical analysis was performed as indicated in the figure legends and text. One-way analysis of variance (ANOVA) was performed to determine the statistical difference between the means of each treatment for experiments in which multiple treatments were compared. Bonferroni’s multiple comparison post-test was combined with ANOVA to compare differences between the means within an experiment.

## Results

### Expression of *MT1* mRNA in the submandibular glands and other organs of embryonic mice

**Fig 1:**
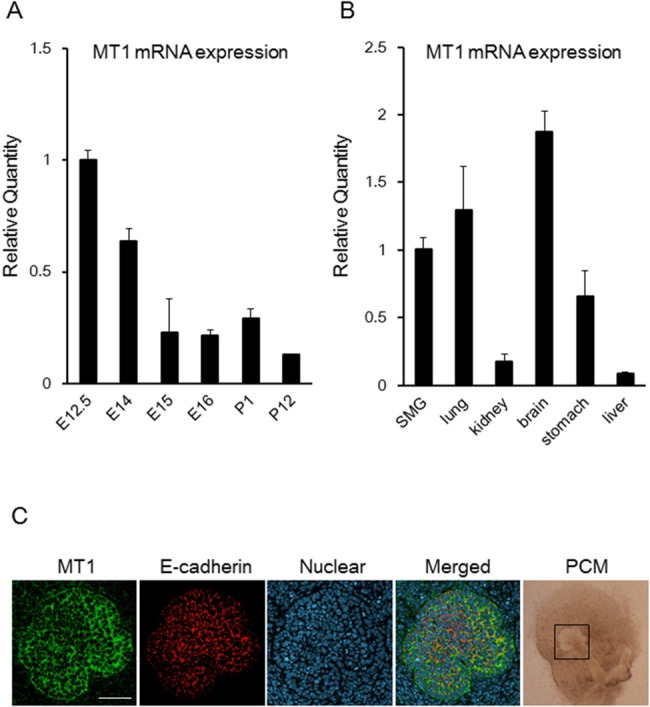
*MT1* mRNA Expression and localization of MT1 and E-cadherin in the submandibular gland of the embryonic mouse. The expression of *MT1* mRNA was determined by qPCR analysis (A, B). *MT1* mRNA expression levels in E14, E15, E16, P1, and P12 were normalized to that in E12.5 (A). *MT1* mRNA expression levels in the lung, kidney, brain, stomach, and liver were normalized to that in the SMG (B). Bars represent the mean ± SEM. MT1 expression during branching morphogenesis was shown by confocal immunofluorescence microscopy imaging of staining for MT1, E-cadherin, or nuclei. MT1 was expressed at the epithelial plasma membrane (C). The three fluorescence images of the same confocal section are merged. The phase-contrast microscope (PCM) image supports the existence of epithelial buds (open square), indicating the morphology of the whole SMG. Scale bar: 50 μm.

We examined the mRNA expression of *MT1* and *MT2* in the submandibular gland (SMG) at several different developmental stages. qPCR analysis revealed that the level of expression of *MT1* mRNA at E12.5 was significantly higher than that at later stages, such as E14, E15, E16, P1, and P12 (p < 0.01; Fig. 1A). Although the expression of *MT2* mRNA showed the same expression pattern as *MT1* mRNA, it was much lower than that of *MT1* mRNA (data not shown). We also tested the expression of *MT1* mRNA in various other organs. *MT1* mRNA was expressed in various organs at E12.5, and the level in both the lung and brain was high, similar to that in the SMG (Fig. 1B). To identify the target of melatonin in oral tissue, we investigated the localization of MT1 in SMGs by immunohistochemical analysis. We used the localization of E-cadherin, a cell-cell adhesion molecule, to define the shape of epithelial cells. We found that MT1 was expressed at the epithelial plasma membrane of embryonic epithelial bud cells (Fig. 1C).

### Melatonin inhibits branching morphogenesis and reduces the size of the submandibular gland epithelium

**Fig 2:**
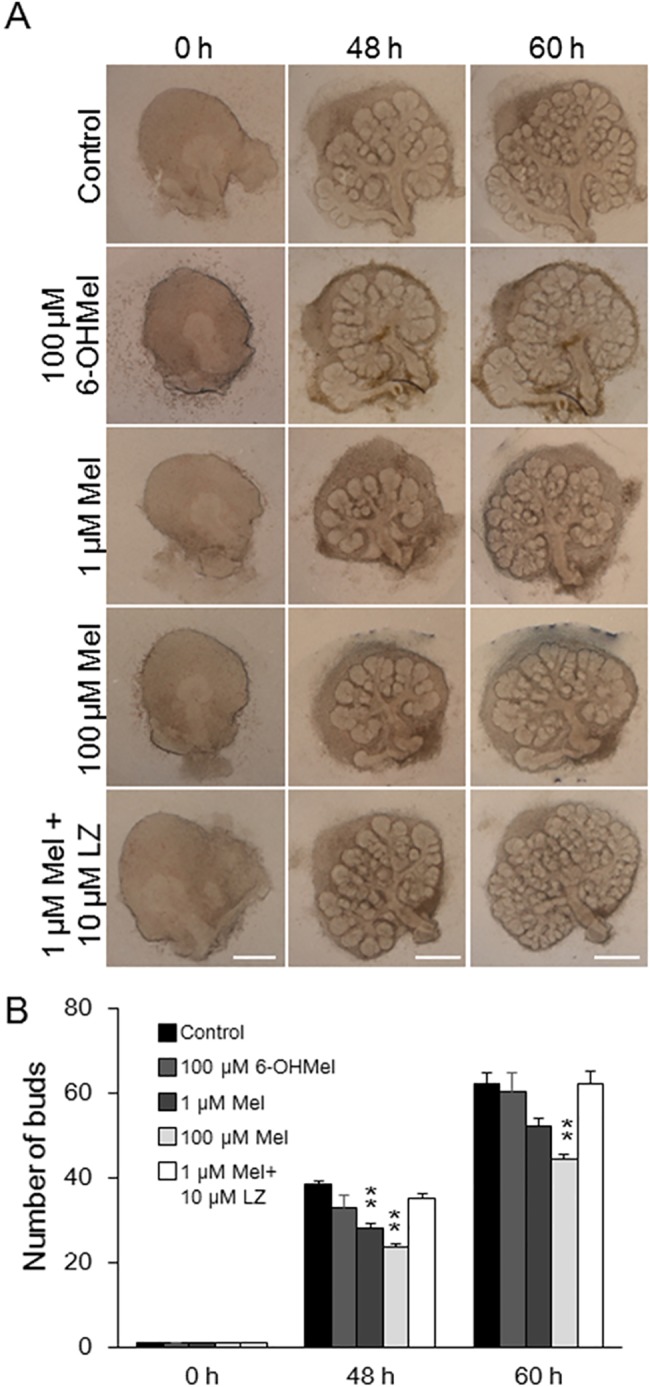
Exogenous melatonin suppresses branching morphogenesis of the submandibular gland. Phase-contrast images show E12.5 SMGs at 0, 48, and 60 h of culture without or with 1 or 100 μM melatonin (Mel), 100 μM 6-OHMel, and 10 μM LZ. Scale bar: 500 μm (A). The effects of treatment with 1 and 100 μM melatonin, 100 μM 6-OHMel, and 10 μM LZ were quantified by counting the number of buds per gland at 0, 48, and 60 h (n = 8) (B). Bars represent the mean ± SEM. **p < 0.01 compared with control.

The expression of the melatonin receptor was high in the early development stages when the salivary gland is highly branched, so we hypothesized that melatonin influences embryonic salivary gland development. We added different doses (1 μM, 100 μM) of exogenous melatonin to the organ culture of E12.5 SMGs from both the ICR (Fig. 2A) and C3H strain (S2 Fig.). The number of lobules in the SMGs was counted after both 48 h and 60 h. The addition of melatonin significantly decreased the number of epithelial branches in the tissue from the ICR mice (p < 0.01; Fig. 2B). Also in the tissue from the C3H mice, the number of buds was decreased (S2 Fig.). This suggested that the response to melatonin might be similar between the ICR and C3H strain of mice (S2 Fig.). We showed that 100 μM 6-hydroxymelatonin (6-OHMel), a bioactive product of melatonin as a negative control, did not affect branching morphogenesis. Luzindole (LZ), a selective melatonin receptor agonist, rescued the branching morphogenesis (Fig. 2).

Growth was also evaluated by measuring the two-dimensional size of the epithelium. We found that the addition of exogenous melatonin slightly inhibited the size of SMG epithelial tissue. We observed a 12~18% of reduction in size (S1 Fig.). These data indicate that melatonin reduces branching morphogenesis and the generation of epithelial tissue in the developing salivary gland.

### Melatonin does not affect proliferation and apoptosis during submandibular gland branching morphogenesis

**Fig 3:**
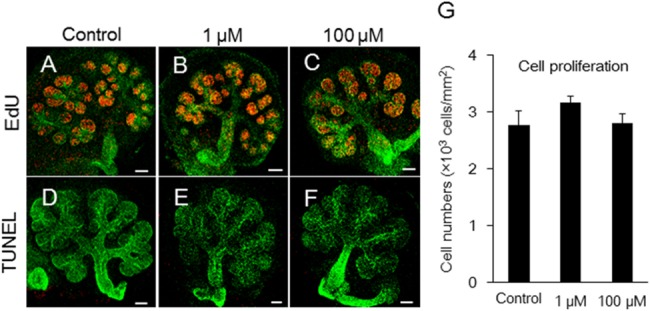
Effects of melatonin on both proliferation and apoptosis during submandibular gland branching morphogenesis. Growing SMG cells in control, 1 μM melatonin, and 100 μM melatonin-treated groups were positive for EdU (A, B, C) and for TUNEL (D, E, F) as markers of cell proliferation and apoptosis, respectively. Proliferating cell nuclei appeared as red punctate spots (A, B, C). Lectin peanut agglutinin (PNA)—Alexa Fluor 647 was used to stain the epithelium green (A-F). Apoptosis of epithelial cells was not detected by TUNEL in red (D, E, F). Scale bar: 100 μm. Fluorescent EdU staining in the epithelium were quantified using ImageJ software (n = 5) (G). The total EdU positive cells were expressed as a ratio of the area. Bars represent the mean ± SEM.

We found that melatonin regulates branching morphogenesis in SMG, but the mechanism by which this occurs remained unclear. To investigate the effect of melatonin on proliferation and on apoptosis, which are cellular processes central to organ development, we performed immunohistochemistry for 5-ethynyl-2′-deoxyuridine (EdU) and terminal deoxynucleotidyl transferase dUTP nick end labeling (TUNEL) after the addition of melatonin to the organ culture medium of E12.5 SMGs for 48 h. Exogenous melatonin (1 μM, 100 μM) did not affect the proliferating EdU-positive cells in the epithelium (Fig. 3A, B). Exogenous melatonin also did not affect apoptosis based on TUNEL staining (Fig. 3A). These data indicate that melatonin inhibits branching morphogenesis independent of proliferation and apoptosis.

### Morphological analysis of melatonin-inhibited branching morphogenesis of the submandibular gland using transmission electron microscopy

**Fig 4:**
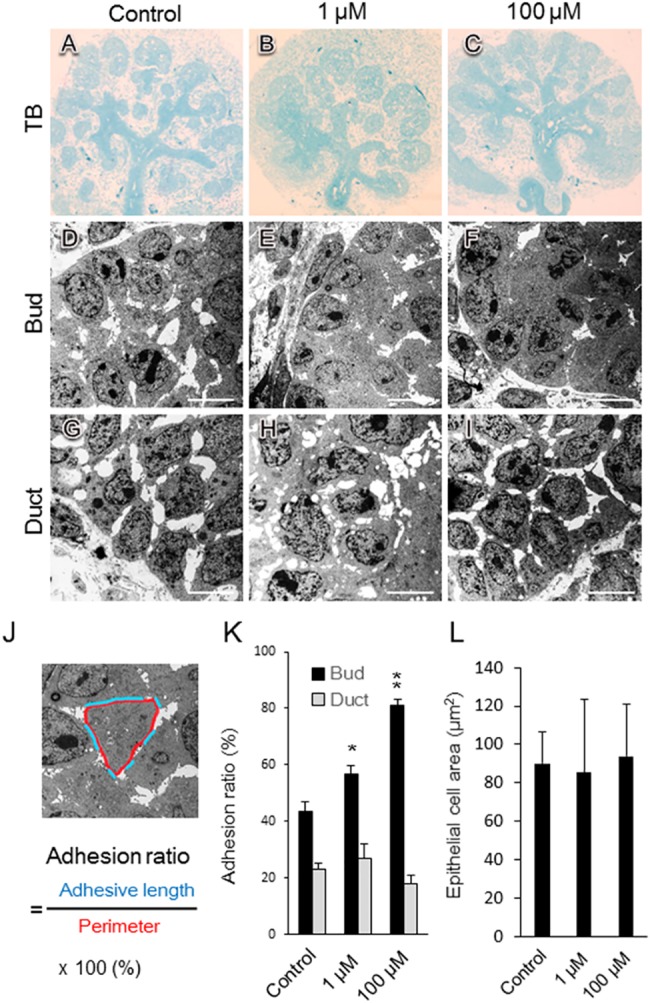
Morphological analysis of melatonin-inhibited branching morphogenesis using transmission electron microscopy. Phase-contrast images show E12.5 SMGs after 48 h of cultured without and with 1 and 100 μM melatonin (A-I). Sections of SMGs were fixed and stained with toluidine blue (A, B, C). Transmission electron microscopy revealed the bud (D, E, F) and duct (G, H, I). The effects of melatonin treatment with both 1 μM (B, E, H) and 100 μM (C, F, I) melatonin were quantified by measuring the adhesion ratio (adhesive length/perimeter × 100%) in SMGs cultured for 48 h. The blue line indicates the adhesive length (length of direct cell-cell contact), and the red line indicates the perimeter. Scale bar: 10 μm (J). The adhesion ratio in buds and ducts treated with either control vehicle, 1 μM melatonin, or 100 μM melatonin was analyzed (bud; n = 10, duct; n = 6) (K). In the buds, the epithelial cell area surrounded in a red line (J) was analyzed using the ImageJ software (n = 8) (L). Bars represent the mean ± SEM. **p < 0.01 compared with control. The experiment was repeated three times using three glands. Data are shown from a representative experiment.

To elucidate the mechanism by which branching morphogenesis is inhibited in the SMG epithelium, we examined the ultrastructure of SMGs cultured for 48 h using transmission electron microscopy (TEM). The epithelial cells of buds cultured with either 1 or 100 μM exogenous melatonin had reduced cytoplasmic projections, and the normal cell-cell contact seemed to decrease. Cells appeared to be in close proximity to each other (Fig. 4D-F).

However, the addition of melatonin did not influence ductal epithelial cells relative to controls (Fig. 4G-I). Direct cell-cell contact was evaluated by measuring the adhesive length/perimeter (adhesion ratio) (Fig. 4J). The adhesion ratio increased in the presence of melatonin (Fig. 4K). However, melatonin did not affect epithelial cell spreading (Fig. 4L). The epithelial cells in the bud changed shape and were more closely packed together in the presence of melatonin, resulting in the inhibition of SMG branching morphogenesis.

### The expression and localization of E-cadherin in presence of melatonin

**Fig 5:**
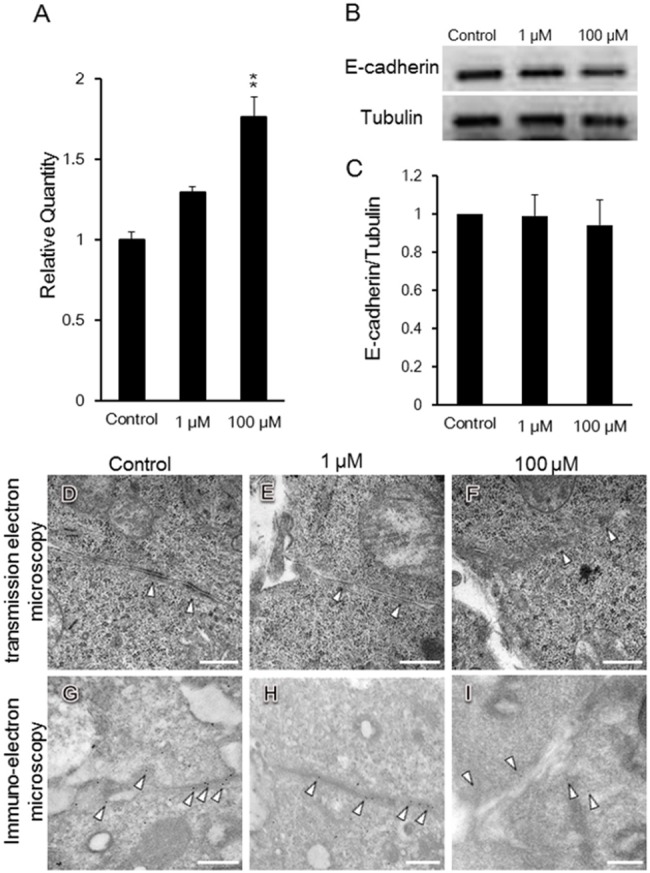
Expression and localization of E-cadherin in the presence of melatonin. *E-cadherin* mRNA expression levels were analyzed. *E-cadherin* mRNA expression levels in SMG cultured with 1 μM and 100 μM melatonin were normalized to that in control SMG without melatonin (n = 3) (A). The expression of E-cadherin protein was analyzed by Western blot. The study was repeated three times, with data shown from a representative experiment (B). The band intensity was quantitated (n = 3) (C). The adherence junction is shown in the presence of melatonin by TEM (D, E, F). The localization of E-cadherin was shown in the presence of melatonin by immuno-electron microscopy (G, H, I). Scale bar: 500 nm. Bars represent the mean ± SEM. **p < 0.01 compared with control.

The expression of *E-cadherin* mRNA slightly increased after 60 h in the presence of melatonin (Fig. 5A). Other cell-cell adhesion molecule, *Occludin* mRNA expression was not changed by melatonin (S3 Fig.). We examined expression of E-cadherin protein by Western blot analysis in the presence of melatonin (Fig. 5B) and quantitated these results (Fig. 5C). The expression of E-cadherin protein did not increase (Fig. 5B, C). Using TEM, the structure of the cell junctions was disturbed (Fig. 5D, E, F; indicated as arrow head). Moreover, by immuno-electron microscopy E-cadherin labeled with colloidal golds (Fig. 5G, H, I; indicated as arrow heads) were found in the cell adhesion junction in the control and in the 1 μM melatonin treated tissue (Fig. 5G, H). On the contrary, E-cadherin was not localized in the junction of epithelial cells in the presence of 100 μM melatonin (Fig. 5I). These data indicated that melatonin changed the pattern of cell-cell junction. Furthermore, in the presence of melatonin, epithelial cells are more closely packed together, and branching morphogenesis during embryonic development is inhibited.

### Production of melatonin by embryonic submandibular glands

**Fig 6:**
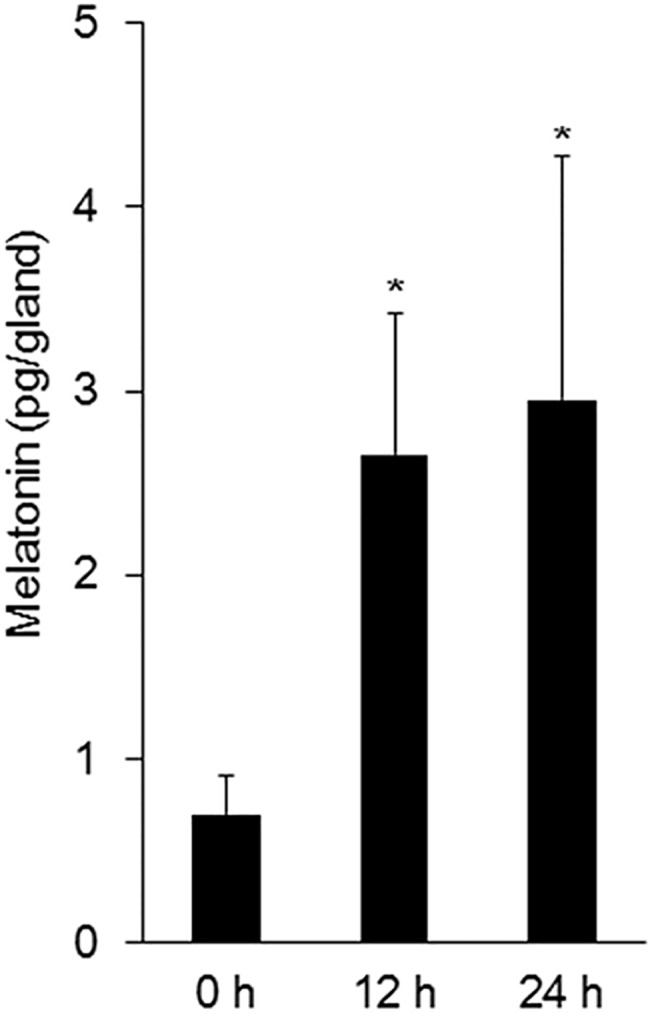
Production of melatonin by embryonic submandibular glands. In C3H mice, the total amount of melatonin in the SMGs and culture medium are shown. Bars represent the mean ± SEM. *p < 0.05 compared with control.

The amount of melatonin in E12.5 SMGs of C3H mice was 0.70 ± 0.10 pg/gland (n = 4). No melatonin was detected in adult SMGs. E12.5 SMGs were grown in medium for 12h and 24 h. The total amount of melatonin in the embryonic SMGs and culture medium was 2.66 ± 0.39 pg/gland (cultured for 12 h, n = 4) and 2.95 ± 0.66 (cultured for 24 h, n = 4) (Fig. 6). The data suggest that the amount of melatonin statistically increased in a time-dependent manner in organ culture (p < 0.05).

## Discussion

Several studies have reported that melatonin affects the formation of bone [32], teeth [21], testes [1], and adrenal glands [33]. There are a few developmental studies on the role of melatonin. Pregnant mice given melatonin had male mice that had a reduction in testicular weight at 5 weeks. Their testicular weights were significantly less than that of control mice [1]. However, there is no embryonic studies involving melatonin. Melatonin crosses the placental barrier [34]. Maternal melatonin is responsible for the plasma concentration in the fetus. It was not investigated whether embryo expresses melatonin. The plasma of both adult humans and mice contains about 1 nM melatonin (physiological level) [35] [36]. Another study showed that 10 nM and 100 nM were considered physiological concentration of melatonin [37]. Our experiments used relatively high melatonin concentrations (1 μM and 100 μM), which may not be physiologically relevant. In previous research, chronic melatonin at lower concentrations (10 nM to 10 μM: for 14 days) and acute melatonin at higher concentrations (100 μM to 2 mM: for 48 h) were found to inhibit the growth and viability of prostate cancer cells [38]. We have investigated the pharmacological effect of acute melatonin at high concentration in organ culture of ICR mice (melatonin deficient mice).

Several studies have demonstrated melatonin expression in mice. It was reported that many strains of laboratory mice are deficient in melatonin [1], however, melatonin receptors (MT1 and MT2) are expressed in many peripheral organs as well as in the brain [7,39]. ICR mice were also reported as deficient in melatonin. However, we found that MT1 was expressed on the surface of epithelial cells in the embryonic SMGs. Moreover, the expression of *MT1* mRNA at E12.5 was higher than that in the postnatal stage. Since these data support the importance of melatonin in early development of SMG, we investigated the effect of exogenous melatonin on the branching morphogenesis of embryonic SMG in melatonin-deficient ICR mice.

**Fig 7:**
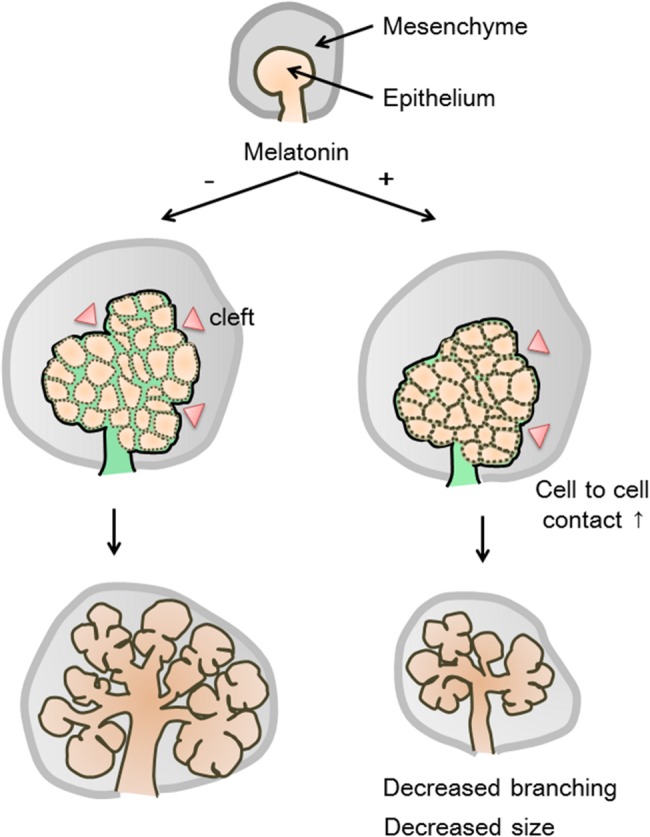
Mechanism of melatonin-inhibited branching morphogenesis in the embryonic submandibular gland. Melatonin changes the morphology of epithelial cells with decreased projections in epithelial cells of the bud. Melatonin increases the direct epithelial cell-cell contacts. The cells are more closely packed and there is less apace between cells. As a result, the size of the organ and the activity of branching morphogenesis are reduced.

Embryonic organs, such as the lung, kidney, and salivary gland, are formed by repeated cycles of epithelial branching morphogenesis. Recent studies indicate that many molecules, including fibronectin, E-cadherin, and BTB (POZ) domain-containing 7 (Btbd7), are involved in this process [40]. In this study, *E-cadherin* mRNA levels were slightly upregulated by the addition of melatonin. However, the expression levels of E-cadherin protein were not increased. E-cadherin expression was disturbed in cell-cell adherence junction as observed using immuno-electron microscopy. Melatonin induced E-cadherin and cell adhesion in MCF-7 human breast cancer cells [41]. E-cadherin is an epithelial adhesion molecule. During early development of the salivary gland, local down-regulation of E-cadherin in epithelial tissues induces cleft formation and branching morphogenesis [26,40]. Our data showed that melatonin increased direct cell-cell contacts and changed the pattern of adherence junctions. Therefore, epithelial cells were closely packed and cleft formation was inhibited with less space between the cells due to this peculiar type of cell junction (Fig. 7). Moreover, melatonin might affect other branching organs such as lung and kidney, which expressed *MT1* mRNA.

Finally, we investigated whether the embryonic SMGs in melatonin-proficient C3H mice would produce and secrete melatonin. To the best of our knowledge, ours is the first report to demonstrate that embryonic SMGs produce melatonin. Recently, it was proposed that melatonin, acting as an autocrine or paracrine factor in lymphoid cells, could be involved in regulating the human immune system [42]. The melatonin secreted by embryonic salivary glands could act in a similar manner to influence organ development. In early embryonic stages, melatonin may maintain the form of the salivary gland until the initiation of branching morphogenesis. Furthermore, the circadian rhythm of melatonin may influence the development of salivary glands. In mammals, the circadian pacemaker in the SCN regulates pineal melatonin rhythms in response to photic signals using adrenergic signals [43]. However, it is unclear whether melatonin production by peripheral organs has a circadian rhythm. We have not yet investigated the rhythm of melatonin production by the salivary gland. If there is a circadian rhythm of melatonin production in the salivary gland, branching morphogenesis could occur during periods of low melatonin concentration in the early stages of gland formation.

## Conclusions

We have shown that melatonin receptors are expressed in embryonic salivary glands, specifically during salivary gland branching morphogenesis. Melatonin inhibits branching morphogenesis independent of both apoptosis and cell proliferation. Furthermore, TEM revealed a possible novel mechanism of melatonin-inhibited branching morphogenesis through epithelial cell-cell adhesion. We speculate that melatonin may be a “brake” for branching morphogenesis acting prior to initiation of branching to maintain the tissue and that its loss and loss of its receptors may trigger branching morphogenesis.

## Supporting Information

S1 Fig Exogenous melatonin reduces the epithelial area of the submandibular gland. Phase-contrast images show E12.5 SMGs at 0, 48, and 60 h of culture without or with 1 or 100 μM melatonin (Mel), 100 μM 6-hydroxymelatonin (6-OHMel), and 10 μM luzindole (LZ). Scale bar: 500 μm (A). The effects of treatment with 1 and 100 μM melatonin were quantified by measuring the area of epithelial tissue at 0, 48, and 60 h. White dotted lines (A) indicate epithelial tissue. The major ducts were excluded from the area quantification (n = 8). Melatonin inhibited slightly the size of the epithelial tissue. Reduction of epithelial area; Control vs 1 μM: 12.1% (48 h), 15.2% (60 h); Control vs 100 μM: 15.1% (48 h), 18.4% (60 h) (B). Bars represent the mean ± SEM. There is no significant difference between control and other samples.
(TIF)

S2 Fig Exogenous melatonin suppresses branching morphogenesis of SMGs in C3H mice. Phase-contrast images show E12.5 SMGs in C3H mice at 0, 48, and 60 h of culture without or with 1 or 100 μM melatonin. Scale bar: 500 μm (A). The effects of treatment with 1 and 100 μM melatonin were quantified by counting the number of buds per gland at 0, 48, and 60 h (n = 8). In C3H mice, the number of buds was decreased (B). In ICR mice, the number of buds was also decreased (C). Bars represent the mean ± SEM. *p < 0.05 compared with control. **p < 0.01 compared with control.
(TIF)

S3 Fig The expression of Occludin in the presence of melatonin. *Occludin* mRNA expression levels were analyzed. *Occludin* mRNA expression levels in SMGs cultured with 1 μM and 100 μM melatonin were normalized to that in control SMGs without melatonin (n = 3). Exogenous melatonin did not affect the mRNA expression of *Occludin*. Bars represent the mean ± SEM. The expression was repeated several times, with data shown from a representative experiment. The primer sequences used were as follows: *Occludin*: 5′-AAGTGAATGGCAAGCGATCATA-3′ and 5′-CTGTACCGAGGCTGCCTGAA-3′.
(TIF)
