# Supplementary figures and images for: Melatonin Inhibits Embryonic Salivary Gland Branching Morphogenesis by Regulating Both Epithelial Cell Adhesion and Morphology
Source: PLoS One. 2015 Apr 15;10(4):e0119960. doi: 10.1371/journal.pone.0119960 (PMC4398443; doi:10.1371/journal.pone.0119960)

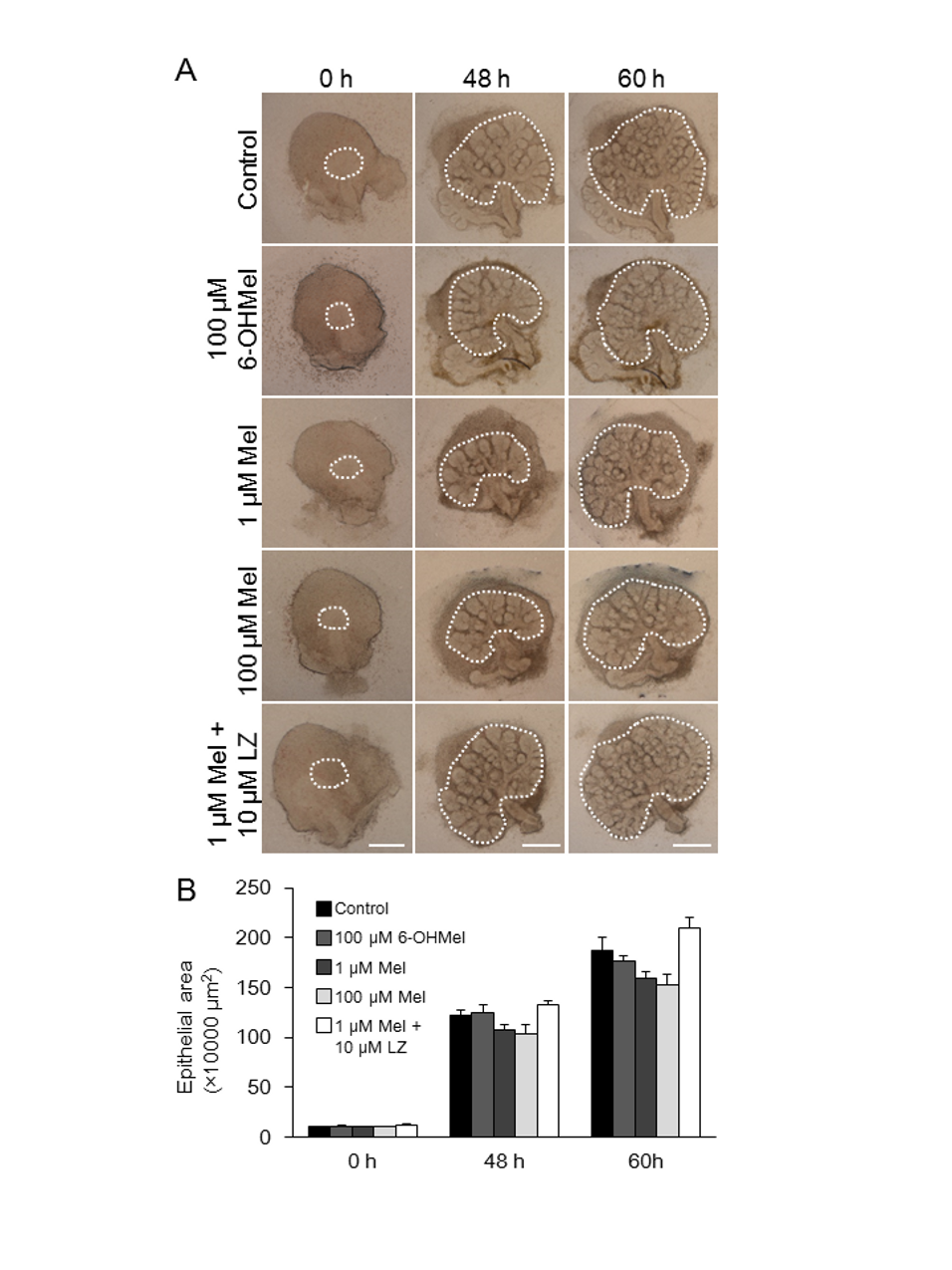

Supplement: S1 Fig — Phase-contrast images show E12.5 SMGs at 0, 48, and 60 h of culture without or with 1 or 100 μM melatonin (Mel), 100 μM 6-hydroxymelatonin (6-OHMel), and 10 μM luzindole (LZ). Scale bar: 500 μm (A). The effects of treatment with 1 and 100 μM melatonin were quantified by measuring the area of epithelial tissue at 0, 48, and 60 h. White dotted lines (A) indicate epithelial tissue. The major ducts were excluded from the area quantification (n = 8). Melatonin inhibited slightly the size of the epithelial tissue. Reduction of epithelial area; Control vs 1 μM: 12.1% (48 h), 15.2% (60 h); Control vs 100 μM: 15.1% (48 h), 18.4% (60 h) (B). Bars represent the mean ± SEM. There is no significant difference between control and other samples. (TIF) [file pone.0119960.s001.tif]

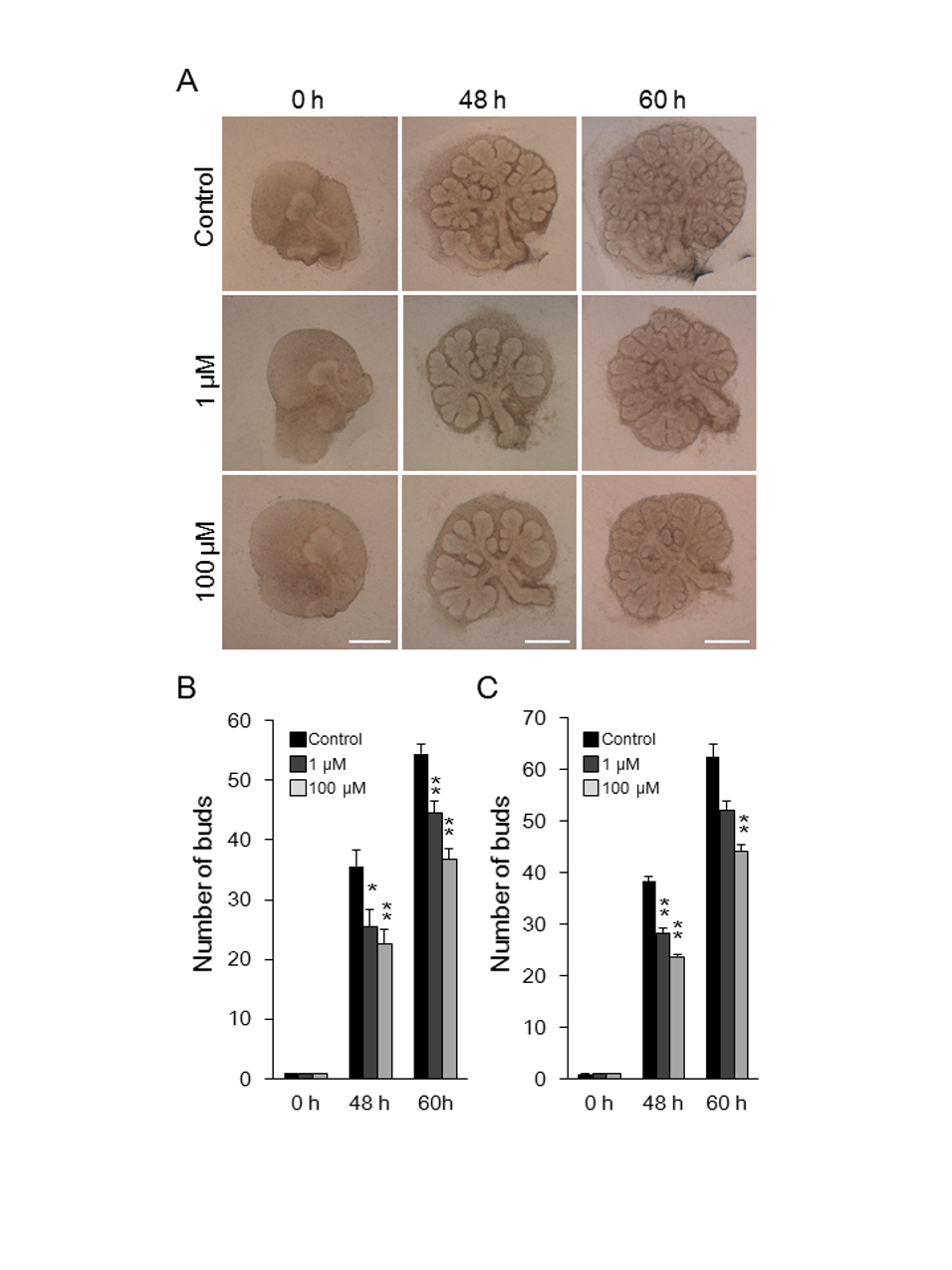

Supplement: S2 Fig — Phase-contrast images show E12.5 SMGs in C3H mice at 0, 48, and 60 h of culture without or with 1 or 100 μM melatonin. Scale bar: 500 μm (A). The effects of treatment with 1 and 100 μM melatonin were quantified by counting the number of buds per gland at 0, 48, and 60 h (n = 8). In C3H mice, the number of buds was decreased (B). In ICR mice, the number of buds was also decreased (C). Bars represent the mean ± SEM. *p < 0.05 compared with control. **p < 0.01 compared with control. (TIF) [file pone.0119960.s002.tif]

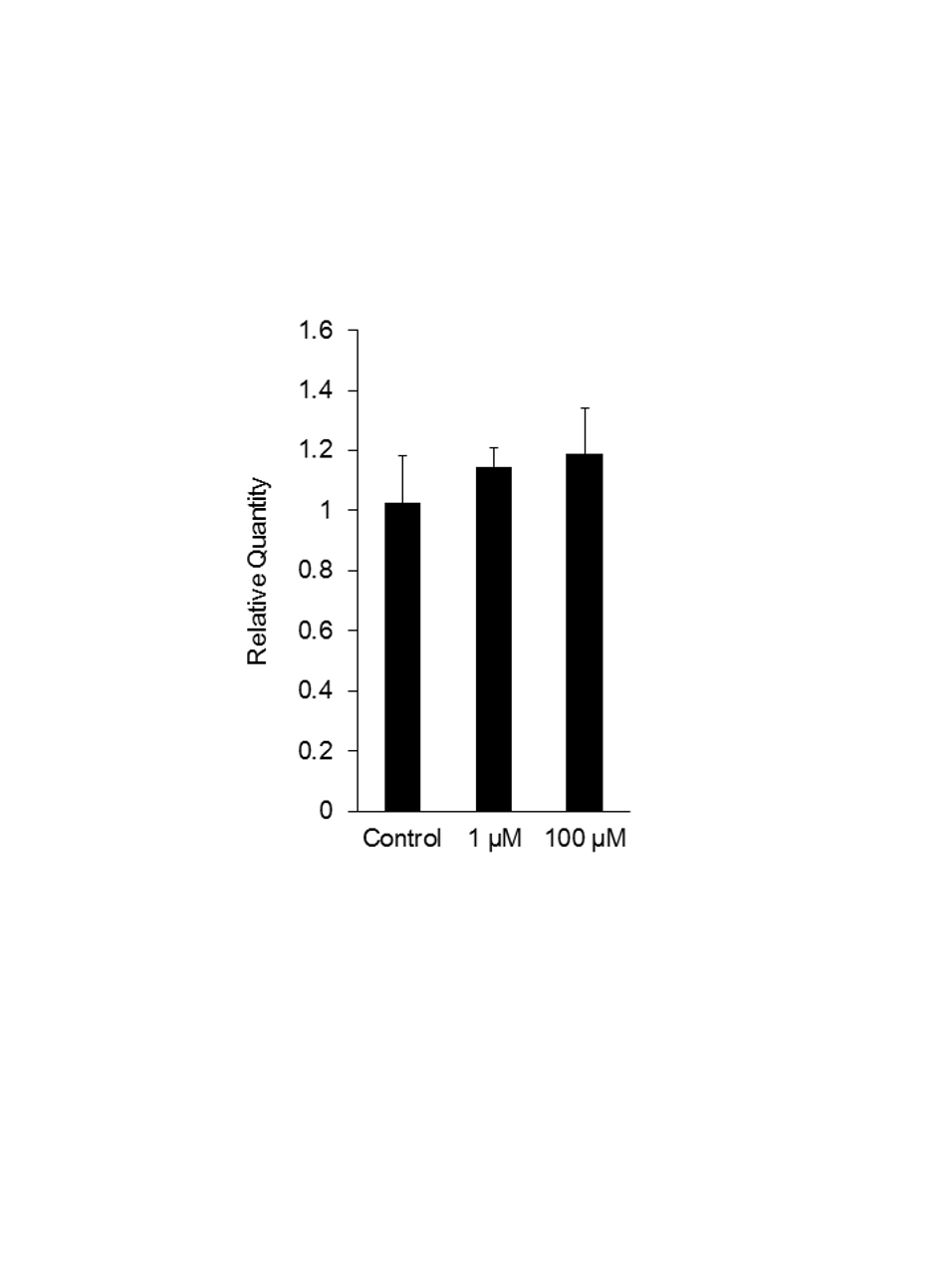

Supplement: S3 Fig — Occludin mRNA expression levels were analyzed. Occludin mRNA expression levels in SMGs cultured with 1 μM and 100 μM melatonin were normalized to that in control SMGs without melatonin (n = 3). Exogenous melatonin did not affect the mRNA expression of Occludin. Bars represent the mean ± SEM. The expression was repeated several times, with data shown from a representative experiment. The primer sequences used were as follows: Occludin: 5′-AAGTGAATGGCAAGCGATCATA-3′ and 5′-CTGTACCGAGGCTGCCTGAA-3′. (TIF) [file pone.0119960.s003.tif]
